# Use of short tandem repeat fingerprinting to validate sample origins in hepatitis C virus molecular epidemiology studies

**DOI:** 10.1099/vir.0.057828-0

**Published:** 2014-01

**Authors:** Victoria C. Edwards, C. Patrick McClure, Richard J. P. Brown, Emma Thompson, William L. Irving, Jonathan K. Ball

**Affiliations:** 1 School of Molecular Medical Sciences, University of Nottingham, Queen’s Medical Centre, Nottingham, UK; 2 Nottingham Digestive Diseases Centre Biomedical Research Unit, University of Nottingham, Queen’s Medical Centre, Nottingham, UK; 3 MRC-University of Glasgow Centre for Virus Research, Garscube Campus, Glasgow, UK

## Abstract

Sequence analysis is used to define the molecular epidemiology and evolution of the hepatitis C virus. Whilst most studies have shown that individual patients harbour viruses that are derived from a limited number of highly related strains, some recent reports have shown that some patients can be co-infected with very distinct variants whose frequency can fluctuate greatly. Whilst co-infection with highly divergent strains is possible, an alternative explanation is that such data represent contamination or sample mix-up. In this study, we have shown that DNA fingerprinting techniques can accurately assess sample provenance and differentiate between samples that are truly exhibiting mixed infection from those that harbour distinct virus populations due to sample mix-up. We have argued that this approach should be adopted routinely in virus sequence analyses to validate sample provenance.

Due to the largely asymptomatic nature of acute hepatitis C virus (HCV) infection, identifying individuals during this phase of the disease is problematic. As a result, studies of cohorts of such individuals are invaluable to improve our understanding of virus evolution and its impact on infection outcome. Most studies have shown that infection in humans and animal models is established by a limited number of highly related founder viruses (Brown *et al.*, 2012; Bull *et al.*, 2011; Li *et al.*, 2012). Post-transmission there is a genetic bottleneck characterized by outgrowth of a selected variant (Bull *et al.*, 2011; Wang *et al.*, 2010). However, at least one study has shown that individuals presenting with acute infections demonstrating large fluctuations in viral load are associated with infection with multiple genetically distinct strains (Smith *et al.*, 2010). Whilst such a dynamic flux of viral variants could be due to simultaneous and/or rapid reinfection by distinct viral variants, it is also possible that this phenomenon could be due to contamination or sample mix-up. Given the importance of the studies of virus evolution in early infection and the need to ensure sample provenance in such studies, we assessed whether short tandem repeat (STR) fingerprinting could be used to define the likely origins of serum samples from two cohorts: one set of samples from a cohort of HCV/human immunodeficiency virus-infected men and the other from a cohort of Egyptian healthcare workers from Egypt for whom sample mix-up was suspected.

The Egyptian study cohort consisted of 32 subjects reported to be suffering from acute HCV infection. Sequential samples were available and these were reported to have been collected over a 300-day period spanning the acute phase of infection, including the antibody-negative/RNA-positive window period. Individual subjects were designated a three-letter ID and sequential samples numbered chronologically. A second, smaller, cohort consisted of two patients (designated UK 1 and UK 2), each suspected of harbouring distinct genotypes of HCV at different time points during acute infection. Two sequential samples (designated ‘a’ and ‘b’) taken 1 month apart were available for each patient.

Nucleic acids (RNA and DNA) were extracted from study samples and control samples using a QIAamp MinElute Virus Spin Kit (Qiagen). For amplification of the 5′ non-coding region (NCR), cDNA was generated with random hexamers and 200 U Moloney murine leukaemia virus (MMLV) reverse transcriptase (Fermentas) according to the manufacturer’s instructions. The viral load of the study samples was determined by quantitative PCR (qPCR) of the 5′ NCR using a gene-specific primer and Scorpion probe. Input cDNA was quantified on an Mx4000 Multiplex Quantitative PCR System (Agilent Technologies) alongside standard controls and results converted to genome copies per millilitre of serum.

For amplification of the first hypervariable region (HVR1) of the HCV E2 glycoprotein, cDNA was generated from control samples using the genotype 4-specific primer OAS4M (5′-CAC CAG CGG CTG AAG CAG CAT TGA-3′) or the genotype 1-specific primer OAS1a (5′- GGG ATG CTG CAT TGA GTA-3′) with 15 U ThermoScript reverse transcriptase (Invitrogen) and 8.5 µl RNA according to the manufacturer’s instructions. For the study samples, cDNA was generated with random hexamers and 200 U MMLV reverse transcriptase according to the manufacturer’s instructions. A 270 bp fragment corresponding to HVR1 of E2 and the E1 and E2 flanking regions was amplified in a nested PCR using genotype-specific primers. For genotype 1: first round, EOS (5′-GGA CGG GGT AAA CTA TGC AAC AGG-3′) and OAS1a; second round, 170gt1 (5′-CAC CAT GGG TTG CTC TTT CTC TAT C-3′) and IASGT1 (5′-TTA CGC CTC CGC TTG GGA TAT GAG TAA CAT CAT-3′). For genotype 4: first round, EOS and E10A (5′-TCA TTG CAG TTC AGG GCA GTC CTG TTG ATG-3′); second round, EIIS_MOD (5′-TGG GAT ATG ATG ATG AAC TGG-3′) and EIIA (5′-CTG TTG ATG TGC CAG CTG CCA-3′).

PCR-positive samples were purified and sequenced. All available sequences were aligned using mega4 software (Tamura *et al.*, 2007) and the evolutionary relationship inferred using the neighbour-joining method (Saitou & Nei, 1987).

STR analysis was carried out on serum-extracted nucleic acid samples using three separate loci. Each STR was amplified in a separate PCR from 5 µl RNA using 0.3 U HotStarTaq DNA polymerase (Qiagen) and gene-specific primers: TH01 F and TH01 R, vWA F and vWA R, and D21S11 F and D21S11 R (Opel *et al.*, 2007), according to the manufacturer’s instructions. The sense primer in each pair was conjugated to a different fluorophore. PCR products were mixed and run alongside the GeneScan 500 LIZ Size Standard (Applied Biosystems) on an ABI Prism 3130 fluorescent DNA analyser (PerkinElmer). Peak traces were analysed using Peak Scanner 1.0 software (Applied Biosystems) to determine the size (in base pairs) of the allele(s) at each locus within all samples. Cluster analysis of STR allele sizes was carried out using GeneMarker v2.2.2 software (Softgenetics) to generate a distance matrix based on the Euclidean distance between single samples.

The Egyptian study cohort and UK cohort were sampled longitudinally during early acute infection. Several patients in the Egyptian study cohort were found to exhibit ‘yo-yo’ viral load dynamics (Table S1, available in JGV Online), which has been linked to fluctuations in the complexity of the virus population (Smith *et al.*, 2010). Phylogenetic analysis of HVR1 sequences isolated from the study cohort alongside reference genotype sequences showed that the majority of patients were infected with a genotype 4 virus (Fig. 1a). A small number of viral sequences clustered most closely to genotype 1 reference strains. HCV genotype 4 is the most prevalent genotype within the Egyptian population (Dusheiko *et al.*, 1994; Ramia & Eid-Fares, 2006). Patient UK 2 was co-infected with genotype 1a and genotype 4d viruses, as had been shown previously (unpublished). However, patient UK 1 contained only genotype 1 viral sequences (Fig. 1a), although previous analyses of these samples showed co-infection with genotype 1a and genotype 4d viruses (unpublished).

**Fig. 1.**
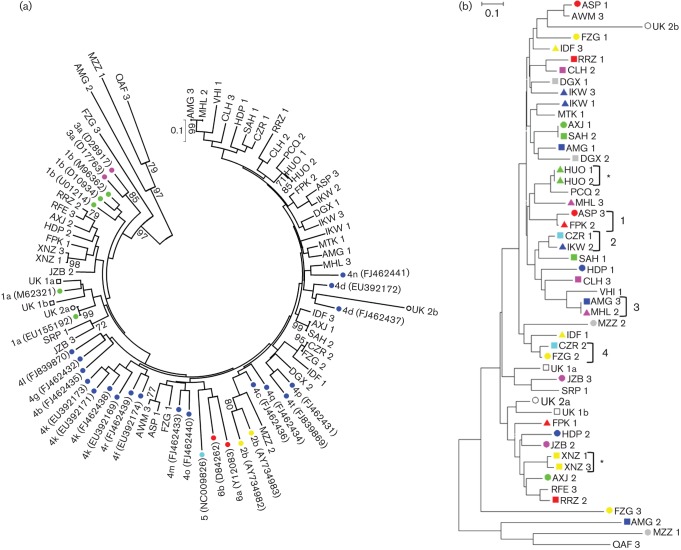
Genotyping viral isolates based on the 270 bp region encompassing HVR1 of E2 and the E1 and E2 flanking regions. Evolutionary history was inferred using the neighbour-joining method and evolutionary distances computed using the maximum-composite-likelihood method. Percentage bootstrap support from 500 replicates is shown (only values greater than 70 % are shown). (a) Genotype was assessed by alignment to reference genotype sequences, highlighted by coloured circles: green, genotype 1; yellow, genotype 2; pink, genotype 3; dark blue, genotype 4; light blue, genotype 5; red, genotype 6. Samples UK 1a, UK 1b, UK 2a and UK 2b are included. Study samples are listed by their three-letter/single-digit ID code. GenBank accession numbers are given in parentheses. (b) Cluster analysis of patient-derived viral isolates and control samples based on HVR1 sequence alignment. Study samples are highlighted with coloured shapes according to patient ID to aid the identification of patient clusters. Patients for whom only one sample contained nucleic acid have been left blank. UK samples are highlighted by unfilled squares (patient UK 1) or circles (patient UK 2). Samples that match according to patient ID are highlighted by an asterisk. Clusters of interest are numbered 1–4.

HVR1 sequences derived from the Egyptian study and UK cohort samples were subjected to further phylogenetic analysis to determine epidemiological relationships (Fig. 1b). In the absence of mixed infection, viral sequences that are epidemiologically linked, i.e. derived from a single patient during the acute phase of infection, would be expected to cluster closely on a phylogenetic tree. Surprisingly, the majority of Egyptian patient samples were distributed throughout the tree. Sequential samples from only two of the patients, HUO and XNZ, harboured HVR1 sequences that clustered on the tree (indicated by an asterisk, Fig. 1b). Similarly, virus sequences derived from the UK samples UK 1a and UK 1b, as well as samples UK 2a and UK 2b, did not cluster (Fig. 1b). In a recent study, mixed acute infections were described in which multiple infections with up to three subtypes was observed. These infections were also characterized by highly divergent clades within a single subtype and multiple switches in the dominant variant present at a specific time point (Smith *et al.*, 2010). Therefore, to ascertain whether our data could be explained by mixed acute infection we further characterized the samples using STR analysis of the genomic DNA present in the sera.

Three discrete STR loci were analysed to define relatedness. This technique is employed commonly in DNA fingerprint analysis for forensic purposes (Butler, 2006; Butler *et al.*, 2003) and the loci selected show good discriminatory power in an Egyptian population (Ahmed *et al.*, 2001; Klintschar *et al.*, 1999). Samples derived from the same individual should have identical patterns of STR locus size and therefore cluster together; however, this was not true of this cohort (Fig. 2). The majority of study samples did not cluster according to patient ID and therefore were not derived from a single individual. Importantly, the UK cohort samples did cluster based on STR analysis, demonstrating that each pair of samples was derived from a single patient and confirming the existence of mixed infection. Interestingly, the two Egyptian study samples in which virus clustered closely (HUO and XNZ, Fig. 1b) also clustered based on STR analysis (Fig. 2), confirming the epidemiological relatedness of these samples.

**Fig. 2.**
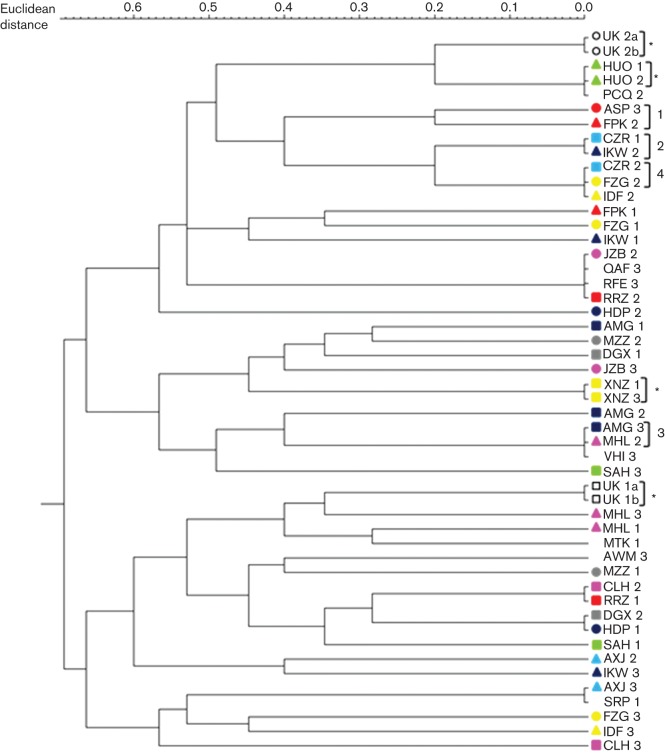
Genotyping patient serum samples using cluster analysis of STR loci. Three STR loci were amplified from serum-extracted DNA samples and STR size determined by examination of peak traces on Peak Scanner 1.0 software. Cluster analysis was carried out using GeneMarker v2.4.0 software to generate a distance matrix based on the Euclidean distance between single samples. Study samples are highlighted with coloured shapes according to patient ID to aid the identification of patient clusters. Patients for whom only one sample contained nucleic acid have been left blank. UK cohort samples are highlighted by unfilled squares (patient UK 1) or circles (patient UK 2). The same colours have been used in Figs 1(b) and 2 to aid comparison of the data. Samples that match according to patient ID are highlighted by an asterisk. Clusters of interest are numbered 1–4.

Further analysis of clustering data (Figs 1a and 2) identified some common groupings in both trees. These included cluster 1 (ASP3, FPK2), cluster 2 (CZR1, IKW2), cluster 3 (AMG3, MHL2) and cluster 4 (CZR2, FZG2). The clustering of these samples based on HVR1 sequences and STR loci suggests that they were linked epidemiologically, i.e. derived from a common source; however, it would be necessary to analyse more STR loci in order to confirm that samples were obtained from a single individual. The use of three STR loci is sufficient to determine that two or more samples are unrelated, but greater discriminating power would be required to definitively prove a relationship between samples.

This analysis was concerned with identifying the relatedness of sequential samples and did not look specifically at within-sample contamination. However, previous analyses using these STR loci have shown that as little as 100 pg (~30 copies) template DNA can be amplified using this method (Opel *et al.*, 2007). Furthermore, mixing experiments have demonstrated that a minor contaminating DNA template, present at only 5–10 % of the major DNA template, can be detected and distinguished successfully from the major DNA template (Gross *et al.*, 2008; Opel *et al.*, 2007). Only two of the Egyptian study samples were found to contain three peaks for an individual locus (data not shown), suggesting that there may have been contaminating DNA within these samples. All other samples, however, appeared to contain a single DNA template.

This study provides an important proof-of-principle that STR fingerprinting can be applied to patient-derived samples to test their provenance. This protocol enabled us to confirm the presence of mixed acute infection in samples obtained from our small UK cohort. Patient UK 1 was infected with two highly divergent genotype 1a strains and patient UK 2 switched from a dominant genotype 1a to a 4d virus. Most importantly, we were able to demonstrate that an extensive sample mix-up had occurred in the Egyptian patient cohort, resulting in the appearance of fluctuating viral load and mixed acute infection in these samples.

In summary, this study has shown that STR analysis can determine the provenance of serum samples used for HCV molecular epidemiology studies.
